# Dynamic Computational Theory Construction and Simulation for the Dynamic Relationship Between Challenge Stressors and Organizational Citizenship Behaviors

**DOI:** 10.3389/fpsyg.2022.891016

**Published:** 2022-07-05

**Authors:** Long Chen, Li Zhang, Qiong Bu

**Affiliations:** ^1^Business School, Hohai University, Nanjing, China; ^2^School of Management, Harbin Institute of Technology, Harbin, China; ^3^China Business Executives Academy, Dalian, China

**Keywords:** challenge stressors, organizational citizenship behavior, system dynamics model, cybernetic theory of stress, social exchange theory

## Abstract

This study explores the dynamic feature of organizational citizenship behaviors under the condition of challenge stressors, as this has not been addressed by previous research. Combining the cybernetic theory of stress and social exchange theory, this study builds a dynamic computational model regarding the circular causality between challenge stressors and organizational citizenship behaviors. By conducting a series of simulation experiments, we validated and demonstrated important questions regarding organizational citizenship behaviors. Specifically, when both the initial value of challenge stressors and the importance of challenge appraisal are higher, organizational citizenship behaviors tend to show a sharped inverted U shape (i.e., organizational citizenship behaviors increase and decrease rapidly) at the early stage. When both the equilibrium level of job satisfaction and the initial value of challenge stressors are higher, organizational citizenship behaviors will show an inverted N shape over time. The number and frequency of assigned challenge tasks have an interactive effect on the accumulation of organizational citizenship behaviors within a period. Our theory contributes to identifying the dynamic relationship between challenge stressors and organizational citizenship behaviors. Findings from dynamic computational theory can offer suggestions for managers to encourage employees’ engagement in organizational citizenship behaviors.

## Introduction

Organizational citizenship behavior (OCB), which refers to a discretionary behavior that goes beyond the duty required in the regular performance evaluation (Van Dyne et al., 1994), has been recognized by scholars and practitioners as an important factor in improving organizational effectiveness (Podsakoff and MacKenzie, 1997). Accordingly, growing studies have focused on the antecedents that encourage employees’ engagement in OCB (e.g., Pooja et al., 2016; Pletzer, 2021; Haldorai et al., 2022). Among these studies, considerable scholars have explored the impact of work stressors on OCB (e.g., Rodell and Judge, 2009; Wallace et al., 2009; Eatough et al., 2011; Zhang et al., 2014; Montani and Dagenais-Desmarais, 2018; Khliefat et al., 2021; Haldorai et al., 2022) because work stressors can consume the resource needed to engage in OCB (Pooja et al., 2016; Montani and Dagenais-Desmarais, 2018). To data, amounts of knowledge have been accumulated on the unidirectional influence of work stressors on OCB. However, previous research has not yet considered the reciprocal relationship between work stressors and OCB occurring over time. This is unfortunate because both OCB and work stressors are dynamic (Rosen et al., 2020; Smith et al., 2020; Lowery et al., 2021; Parke et al., 2021).

This study seeks to address this gap by building a dynamic computational theory that shows the dynamic relationship between challenge stressors and OCBs over time. Challenge stressor refers to “work-related demands or circumstances that, although potentially stressful, may have possible gains for individuals” (Cavanaugh et al., 2000, p. 68). On the one hand, challenge stressor (e.g., workload and time pressure) has become a ubiquitous condition in the workplace (Cavanaugh et al., 2000; Lam et al., 2015). On the other hand, literature on OCB often highlights the role of challenge stressors (e.g., Montani and Dagenais-Desmarais, 2018; Khliefat et al., 2021; Haldorai et al., 2022). These studies have not yet reached a consensus on the relationship between challenge stressors and OCBs. Some studies indicated that the relationship between challenge stressors and OCBs was positive (e.g., Rodell and Judge, 2009) and some others reported a negative relationship (e.g., Khliefat et al., 2021). Nevertheless, most of these studies found an insignificant relationship or even a curvilinear effect (e.g., Wallace et al., 2009; Eatough et al., 2011; Mazzola and Ryan, 2019; Haldorai et al., 2022). However, the focus of our study is not to identify the boundary conditions for these mixed findings but to reveal the dynamic nature of OCB when the challenge stressor changes over time. Furthermore, challenge stressor has the potential to promote OCB because challenge stressor can improve job satisfaction (Cavanaugh et al., 2000) thereby motivating OCB (Eatough et al., 2011). In contrast, hindrance stressor, which refers to “work-related demands or circumstances that tend to constrain or interfere with an individual’s work achievement and that do not tend to be associated with potential gains for the individual,” is less likely to encourage employees’ engagement in OCB. First, hindrance stressors would inhibit personal growth and reduce job satisfaction (Cavanaugh et al., 2000). Second, a hindrance stressor as a kind of work stressor would deplete the resource required to engage in OCB (Montani and Dagenais-Desmarais, 2018). Empirical research also reported that hindrance stressors would decrease OCB (Khliefat et al., 2021). Hence, it is more likely to observe the dynamic nature of OCB under the condition of challenge stressors compared with hindrance stressors.

When theories speak to how key variables affect each other over time, the recommended method is to collect longitudinal data (e.g., Rogosa, 1988). However, utilizing the longitudinal design to examine the causal processes over time requires complex procedures and analytical methods (Vancouver et al., 2010). Moreover, the internal validity of longitudinal design is less than expected (Vancouver et al., 2010). Hence, organizational researchers advocate establishing dynamic computational theory rather than new methods or statistics (Vancouver et al., 2010; Vancouver and Weinhardt, 2012). Dynamic computational theory refers to the mathematical specification of a theoretical account of how constructs influence each other over time (Davis et al., 2007; Vancouver et al., 2010). Given a set of starting values, we can investigate how key constructs in the dynamic computational theory change over time (Davis et al., 2007). This study will apply dynamic computational theory to demonstrate two functions it might offer to improve the knowledge regarding how OCB changes over time under the condition of dynamic challenge stressors.

First, we used modeling to see whether combining the cybernetic theory of stress (Edwards, 1992) and social exchange theory (Blau, 1964) to demonstrate a series of simple processes with circular causality (e.g., challenge stressor influences OCB, which influences challenge stressor) are viable. The cybernetic theory of stress illustrates (Edwards, 1992) the circular causality of stressor, perception (i.e., perceived stress), coping, and job satisfaction. Vancouver and Weinhardt (2012) indicated that the cybernetic theory of stress could be tested by building a dynamic computational model. However, the cybernetic theory of stress does not account for the features of challenge stressor, such that challenge stressor is associated with challenge appraisal and might foster positive feelings (Cavanaugh et al., 2000; Zhang et al., 2014; Ma et al., 2021). Social exchange theory, which underlies much of the OCB literature (e.g., Ilies et al., 2009; Gong et al., 2010; Ma and Qu, 2011; Roch et al., 2019), argues that employees who are experiencing positive feelings within organizations tend to reciprocate *via* OCB (Blau, 1964; Rodell and Judge, 2009). In addition to this positive causal path from challenge stressor to OCB, there is also a negative causal process that challenge stressor can induce psychological strain and thereby inhibit OCB (Zhang et al., 2014). Hence, we can use this model to reconcile previous inconsistent arguments that appear on the relationship between challenge stressors and OCBs.

Second, our models will consider a basic assumption that people have limited resources (Hobfoll, 1989) and examine what activities an employee prioritizes under the condition of resource depletion. For instance, Eatough et al. (2011) argue that employees may prioritize by reducing discretionary OCB and only focus energy on coping with in-role work tasks because failing to perform the required in-role behavior carries more risks than reducing OCB (Bergeron, 2007). Considering that challenge stressor is a stimulus that places challenging job requirements on employees (LePine et al., 2005), we suggest that employees may give priority to coping with challenge stressors and reduce OCB when having insufficient resources. We will add these assumptions to our dynamic computational model and validate important questions regarding the dynamic relationship between challenge stressors and OCBs.

This study will then elaborate the causal paths and offer details about how we constructed the dynamic computational model by combining the cybernetic theory of stress and social exchange theory. Utilizing previous findings and the phenomenon that our study is committed to elaborating, this study assigns functional expressions for variables and sets the initial value for each parameter. After evaluating the validity of this dynamic computational theory, this study conducts various simulation experiments in different conditions. Based on the results of the simulation experiment, this study puts forward some propositions regarding how OCB changes over time under the condition of dynamic challenge stressors.

## Theory and Dynamic Computational Model

### The Causal Path Based on the Cybernetic Theory of Stress

According to the cybernetic theory of stress, the stimuli from physical and social environments can influence employees’ perceptions, which denotes a non-evaluative subjective representation of any situations and events (Edwards, 1992). Then, employees assert such perception against the desire states which refer to the optimal amounts of acceptability or minimum or maximum acceptable levels that an individual consciously wants (Lord and Hanges, 1987). The discrepancy between perceived states and desire states can influence employees’ job satisfaction (i.e., how an employee feels about their job and its various aspects, Spector, 1997) and coping behavior. The effect of this discrepancy is moderated by the importance of job satisfaction and the importance of coping behavior (Edwards, 1992). Finally, coping behavior will help to improve the physical and social environments. The above theoretical logic is consistent with some stress theories (e.g., Cummings and Gooper, 1979; Edwards, 1992). Therefore, this study can adopt the cybernetic theory of stress to reveal the dynamic characteristics of coping with challenge stressors.

The challenge stressor is associated with challenging job requirements and such stressor can bring potential achievement for the focal employees (Cavanaugh et al., 2000). Examples of challenge stressors are time pressure, job overload, and high levels of job responsibility (McCauley et al., 1994; Zhang et al., 2014). By applying the cybernetic theory of stress to explain the coping process of a challenge stressor, we can obtain the following feedback loops. When employees are faced with a challenge stressor, they should first change their non-evaluative subjective perception of such stressor (i.e., perceived challenge stress). Perceived challenge stress is an indispensable variable because people can respond only when they are aware of external challenge stressors (Mischel, 1973). Then, employees should compare the difference between perceived challenge stress and expected challenge stress which is defined as the maximal level of challenge stressor that an individual expects to undertake. This discrepancy will motivate employees to invest resources into coping with challenge stressors. When perceived challenge stress is higher than the expected challenge stress, employees should make great efforts to reduce challenge stressor. In contrast, when perceived challenge stress is equal to or lower than the expected challenge stress, employees should continue to decrease such challenging job requirements but with less motivation (Edwards, 1992; Vancouver and Weinhardt, 2012). Consistent with Edwards’ cybernetic theory of stress, the effect of stress discrepancy on challenge stressor coping behavior is moderated by the importance of coping behavior (Edwards, 1992).

For the path from challenge stressor to job satisfaction, evidence has indicated that challenge stressor increases strain (LePine et al., 2004; LePine et al., 2005), which includes anxiety, exhaustion, depression, and burnout (Jex, 1998). Evidence has shown that strain has a damaging effect on job satisfaction (Thoresen et al., 2003). Hence, we assume that employees may experience strain, which leads to low job satisfaction when perceived challenge stress is larger than the expected challenge stress. However, employees will feel no strain when perceived challenge stress is less than the expected challenge stress. Similarly, the impact of stress discrepancy on strain is moderated by the importance of strain.

**FIGURE 1:**
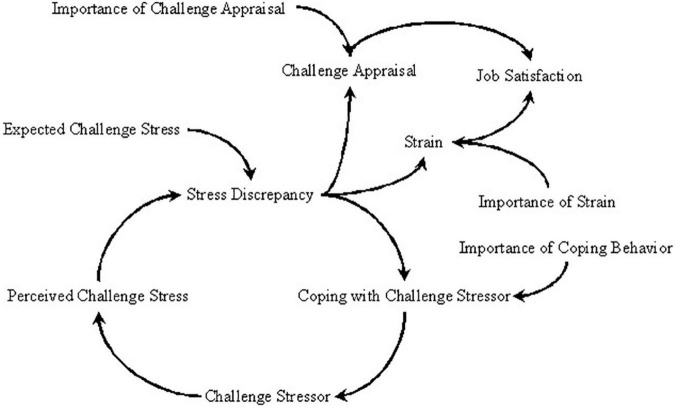
The causal path based on the cybernetic theory of stress.

Finally, challenge appraisal will appear when people suffer from situations perceived as having the potential for personal growth, mastery, and rewards (Skinner and Brewer, 2002). Accordingly, employees have a high tendency to form challenge appraisals when confronting challenge stressors because such stressors can bring potential benefits to employees (Webster et al., 2011). Following this logic, we argue that challenge stressors can improve employees’ job satisfaction by cultivating positive feelings. This theoretical logic has been supported by some empirical studies (e.g., Zhang and Lu, 2009; Webster et al., 2011). For example, Podsakoff et al.’s (2007) study indicated that challenge stressor was positively related to job satisfaction. However, Edwards (1992) cybernetic theory of stress has not considered the positive path from challenge stressor to job satisfaction. This research will fill this gap by adding a positive path to the cybernetic theory of stress. In sum, the challenge stressor coping model based on the cybernetic theory of stress is shown in Figure 1.

### The Causal Path Based on Social Exchange Theory

**FIGURE 2:**
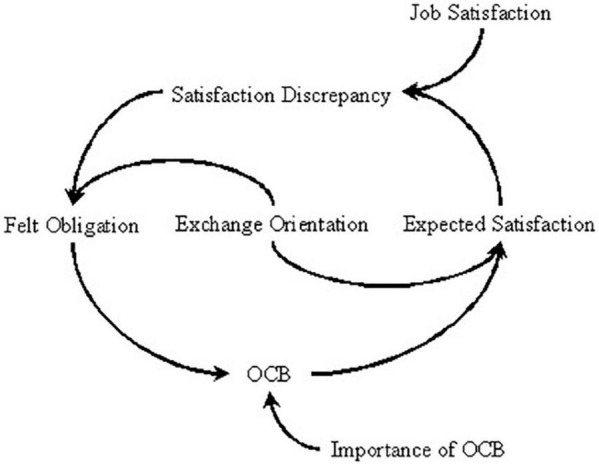
The causal path based on social exchange theory.

Social exchange theory pertains that, when people interact with each other, they should follow the norm of reciprocity (Cropanzano and Mitchell, 2005). Guided by this norm, employees may produce a sense of obligation when employees perceive more positive treatment in organizations than what they expect (Blau, 1964). Felt obligation refers to the belief that an employee is personally obligated to act in ways valued by the organization (Eisenberger et al., 2001). As a result of the felt obligation, we argue that employees will engage in OCB more frequently (Roch et al., 2019). With the increase of OCB, employees will impose a high expectation on organizations and want to experience more positive feelings at work. Therefore, we can obtain the feedback loop in Figure 2 according to the social exchange theory. As shown in Figure 2, we argue that employees should compare job satisfaction and expected satisfaction. Employees are more likely to produce felt obligation when job satisfaction is beyond expectation. Motivated by felt obligation, employees tend to engage in OCB (Roch et al., 2019). According to the norm of reciprocity (Cropanzano and Mitchell, 2005), when engaging in OCB more frequently, employees’ expectation of acquiring positive treatment from their organizations will be higher as well as the expected satisfaction. Nevertheless, the exchange rule that an individual should reciprocate to others after receiving others’ favor and expect others to reciprocate after offering kindness to others is not established for every individual. It is regulated by exchange orientation, which refers to the personal disposition that focuses on the balance of reciprocity (Murstein et al., 1977). Therefore, this study proposes that the relationship between satisfaction discrepancy and felt obligation as well as the relationship between OCB and expected satisfaction is moderated by exchange orientation. In addition, we adopted the importance of OCB as a parameter that can moderate the effect of felt obligation on OCB. By doing so, we have demonstrated that felt obligation does not necessarily lead to OCB.

### An Integrated Causal Path of Challenge-Stressor-Coping

**FIGURE 3:**
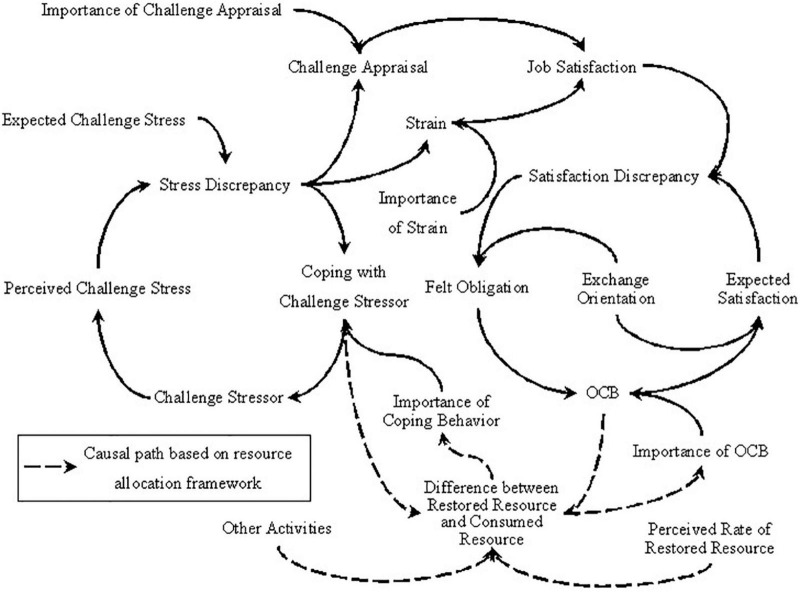
The integrated causal path of copping with challenge stressor.

Integrating the cybernetic theory of stress and social exchange theory, we constructed a model seen in Figure 3. According to this model, challenge stressor has both positive and negative effects on job satisfaction. When job satisfaction is more than expected satisfaction, employees will feel an obligation and invest resources to conduct OCB. The positive discrepancy between the perception of challenge stressors and expected challenge stress motivates employees to engage in coping behavior that is helpful for them to decrease challenge stressors. Employees will continue to cope with challenge stressors with less effort until the source of the challenge stressor disappears. Different from previous research, we add the path from challenge stressor to challenge appraisal. Specifically, when the perceived challenge stress is larger than the expected challenge stress, employees will experience challenge appraisal because challenge stressors can bring accomplishment and personal growth to them (Cavanaugh et al., 2000). Such challenge appraisal has a positive effect on job satisfaction. To exhibit that employees would not always appraise challenge stressors as a challenge, we adopted the importance of challenge appraisal as a parameter that can moderate the relationship between the stress discrepancy and challenge appraisal. Considering that people have limited resources and only restore finite energy within 1 day (Hobfoll, 1989), we believe that an individual can only engage in a limited number of activities. Assumed that the consumed resource is beyond the restored resource, how will the individual clarify the priority between stress coping behavior and OCB? Due to the fact that OCB is an extra-role behavior (Van Dyne et al., 1994), an employee would not be punished or acquire low-performance evaluation even without engaging in such behavior (Bergeron, 2007). As most of the challenge stressors are from in-role job requirements (Cavanaugh et al., 2000), employees are more likely to undertake the risk of low-performance evaluation if they do not fulfill these job requirements (Bergeron, 2007). Therefore, we suggest that employees give priority to coping with challenge stressors rather than performing OCB when employees have inadequate resources. To operationalize these theoretical assumptions, we define that the importance of coping behavior will increase and the importance of OCB will decrease when the resource is increasingly insufficient. Furthermore, this study also includes the activities that employees have to complete. As a result, we regard the perceived difference between the consumed resource and restored resource as the degree of inadequate resource and use the summation of coping with challenge stressors, OCB, and other activities to represent a consumed resource.

## Adding Math Into the Dynamic Computational Model

This study clarified the mathematical relationship among variables before adopting Vensim PLE6.4 software to stimulate the computational model. We determined the mathematical expressions of variables using the conditional function, the integral function, the regression function, and the random function. In general, the dynamic computational model is not sensitive to the parameter and the simulation results tend to be consistent when the structure of the model is stable (Chen et al., 2019). Hence, this study sets the parameters in the model based on previous research. In the next part, this study will offer details about the mathematical relationship and the parameter settings.

A challenge stressor is an external stimulus. This study will observe the dynamic change of the variables in the dynamic computational model by regulating the initial value of the challenge stressor. This process of changing the values of the parameters and observing the results is called sensitivity analysis (Davis et al., 2007). Before the sensitivity analysis, we need to set an initial value for the challenge stressor. After stimulating the dynamic computational model, we found that this model could be activated only if the initial value of the challenge stressor is greater than 0. Although many empirical studies can offer us reference values (e.g., the mean of challenge stressor), we chose the mean of challenge stressor (i.e., 2.71) from Cavanaugh et al.’s (2000) study, because this study is the basis of research on challenge stressor (e.g., LePine et al., 2004; Podsakoff et al., 2007; Prem et al., 2017). Challenge stressor, which is a level variable, will decrease with the increase in coping with challenge stressors. Hence, we used the integral function to represent the challenge stressors:


$$\begin{array}{l} Challeng\;estressor\\ ={\text{INTEG}}^{\text{1}}(-Coping\;with\;Challenge\;Stressor,\;2.71) \end{array}$$

Perceived challenge stress is an auxiliary variable and is directly determined by the value of the challenge stressor. Hence, we used the function expression that perceived challenge stress is equal to challenge stressors. Consistent with Vancouver and Weinhardt’s (2012) computational model, the expected challenge stress is set as zero. The stress discrepancy compares the difference between perceived challenge stress and expected challenge stress. As abovementioned, there are three scenarios of stress discrepancy. The first scenario is that perceived challenge stress is larger than the expected challenge stress. In this case, stress discrepancy is equal to the value in which perceived challenge stress subtracts expected challenge stress. Second, when perceived challenge stress ranges from zero to expected challenge stress, we suggest that employees will cope with challenge stressors with lower motivation (Vancouver and Weinhardt, 2012). To reflect this setting, this study follows the expression of Vancouver and Weinhardt (2012) and divides stress discrepancy by 2. In the third scenario where perceived challenge stress is equal to or lower than zero, the challenge stressor has been eliminated and employees have no motivation to cope with the challenge stressor. As such, the stress discrepancy will stay at the status of “*-Expected Challenge Stress.*” We used the conditional function to represent stress discrepancy:


$$\begin{array}{l} Stress\;Discrepancy\\ =IF\;THEN\;ELSE^{2}(Perceived\;Challenge\;Stress\\ -Expected\;Challenge\;Stress>0,Perceived\;Challenge\;Stress\\ -Expected\;Challenge\;Stress,IF\;THEN\;ELSE\;(Perceived\\ Challenge\;Stress-Expected\;Challenge\;Stress\;>0,\\ (Perceived\;Challenge\;Stress-Expected\;Challenge\;Stress)\;/2,\\ -Expected\;Challenge\;Stress/2) \end{array}$$

Challenge appraisal is determined by both the stress discrepancy and the importance of challenge appraisal. Only when stress discrepancy is larger than zero (i.e., perceived challenge stress is beyond expected challenge stress) can challenge appraisal be formed. The importance of challenge appraisal represents the likelihood of challenge appraisal when the perceived challenge stress is larger than the expected challenge stress. This study adopts the correlation coefficient between a challenge stressor and a challenge appraisal in previous research as the importance of challenge appraisal and sets it as 0.23.^3^ Therefore, we used the following conditional function to represent challenge appraisal:


$$\begin{array}{l} Challenge\;Appraisal\\ =IF\;THEN\;ELSE\;(Stress\;Discrepancy\;>0,\\ Stress\;Discrepancy^{*}Importance\;of\;Challenge\;Appraisal\;,0) \end{array}$$

Strain is determined by both stress discrepancy and the importance of strain. Only when the stress discrepancy is larger than zero, a strain can be formed. The importance of strain represents the likelihood of strain when perceived challenge stress is larger than the expected challenge stress. We set the importance of strain as 0.29, which is the correlation coefficient between challenge stressor and strain in Mazzola and Ryan’s (2019) study. We used the following conditional function to represent strain:


$$\begin{array}{l} Strain=IF\;THEN\;ELSE\;(Stress\;Discrepancy\;>0,\;\\ \;Stress\;Discrepancy^{*}Importance\;of\;Strain\;,0) \end{array}$$

Job satisfaction, which is a level variable, is affected by challenge appraisal and strain. Moreover, following Vancouver and Weinhardt’s (2012) study, we added a negative feedback loop into the functional expression of job satisfaction. This negative feedback loop is based on the opponent-process theory, which argues that there is a counterforce to push the emerging emotion back to the original equilibrium level when an individual experiences an emotion (Landy, 1978). As an individual can hardly maintain high or low levels of emotion all the time, the emerging emotion will inevitably return to its original level over time (Landy, 1978). Consistent with the parameter setting in Vancouver and Weinhardt’s (2012) study, we set the initial equilibrium level and the opponent-process rate of job satisfaction as zero and 0.5, respectively. Hence, we used the integral function to represent job satisfaction:


$$\begin{array}{l} Job\;Satisfaction\\ =INTEG(Challenge\;Appraisal-Strain\\ +(Initial\;Equilibrium\;Level-Job\;Satisfaction{)}^{*}Opponent\\ Process\;Rate,Initial\;Equilibrium\;Level) \end{array}$$

Coping with challenge stressors is determined by stress discrepancy and the importance of coping behavior. As mentioned above, employees will make great efforts to cope with challenge stressors when stress discrepancy is above zero. When perceived challenge stress is above zero but the stress discrepancy is below zero, they engage in coping behavior with less motivation. To reflect these functions, we adopted Vancouver and Weinhardt’s (2012) expression. When stress discrepancy is above zero, the expression “(1 + *Stress Discrepancy*)*Importance of Coping Behavior” is used to delegate coping with challenge stressors. Adding “1” to “*Stress Discrepancy*” is to guarantee continuity. When perceived challenge stress is above zero but the stress discrepancy is below zero, we used the expression “EXP(*Stress Discrepancy*)” to represent coping with challenge stressor. Finally, we set coping with challenge stressors as zero when the perceived challenge stress is equal to or below zero. Therefore, we used the following conditional function to represent coping with challenge stressors:


$$\begin{array}{l} Coping\;with\;Challenge\;Stressor\\ IF\;THEN\;ELSE(Stress\;Discrepancy\text{ > }0,(\text{1}+Stress\\ Discrepancy{)}^{*}Importance\;of\;Coping\;Behavior,IF\;THEN\\ ELSE(Perceived\;challenge\;stress>0,\\ EXP(Stress\;Discrepancy{)}^{*}Importance\;of\;Coping\;Behavior,0) \end{array}$$

We argue that the importance of coping behavior tends to be enhanced when the restored resource is lower than the consumed resource. In contrast, the importance of coping behavior will remain at the initial value when the restored resource is equal to or larger than the consumed resource. We set the initial importance of coping behavior as 0.29, which is the same as the importance of strain because we have stimulated the dynamic computational model many times and found that the results of this model were not sensitive to this value. Therefore, we used the following conditional function to represent the importance of coping behavior:


$$\begin{array}{l} Importance\;of\;Coping\;Behavior\\ =IF\;THEN\;ELSE(Difference\;between\;Restored\;Resource\\ and\;Consumed\;Resource>0,0.29,0.29+0.29^{*}\\ (-Difference\;between\;Restored\;Resource\;and\;Consumed\\ Resource)/(1-Difference\;between\;Restored\;Resource\;and\\ Consumed\;Resource)) \end{array}$$

The difference between restored resources and consumed resources is determined by the perceived rate of restored resources, coping with challenge stressors, OCB, and other actives. As the perceived rate of restored resources and other actives vary randomly from day to day, we used the random function “RANDOM UNIFORM (0, 2, 0.05)” and “RANDOM UNIFORM(0, 1, 0.05)” to represent the perceived rate of restored resource and other actives, respectively. Hence, we used the following integral function to represent the difference between the restored resource and the consumed resource:


$$\begin{array}{l} Difference\;between\;Restored\;Resource\;and\;Consumed\;Resource\\ =INTEG(Perceived\;Rate\;of\;Restored\;Resource\\ -Coping\;With\;Challenge\;Stressor-OCB-Other\;Activities,0) \end{array}$$

Satisfaction discrepancy is equal to the difference between job satisfaction and expected satisfaction when job satisfaction is above the expected satisfaction. Otherwise, satisfaction discrepancy is equal to zero. Therefore, we used the following conditional function to represent satisfaction discrepancy:


$$\begin{array}{l} Satisfaction\;Discrepancy=\text{IF THEN ELSE}\\ Job\;Satisfaction-Expected\;Satisfaction>\;0,Job\;Satisfaction\\ -Expected\;Satisfaction,0) \end{array}$$

Felt obligation is determined by the satisfaction discrepancy and exchange orientation. We set the exchange orientation as 0.51, which is the correlation coefficient between job satisfaction and felt obligation in Wells et al. (2007) study. As such, we used the following expression to represent felt obligation:


$$\begin{array}{l} Felt\;Obligation\\ -Satisfaction\;Discrepancy^{*}Exchange\;Orientation \end{array}$$

OCB is the product of felt obligation and the importance of OCB. We argue that the importance of OCB tends to be weakened when the restored resource is lower than the consumed resource. In the other case, the importance of OCB will remain at the initial value. We set the initial importance of OCB as 0.2 which is the correlation coefficient between felt obligation and OCB in Roch et al. (2019) study. The following conditional function is to represent the importance of OCB:


$$\begin{array}{l} Importance\;of\;OCB=IF\;THEN\;ELSE\\ (Difference\;between\;Restored\;Resource\;and\;Consumed\\ Resource>0,0.2,0.2/(1-Difference\;between\;Restored\\ Resource\;and\;Consumed\;Resource)) \end{array}$$

The expected satisfaction is a level variable and is determined by OCB and exchange orientation. We suggest that employees tend to expect more job satisfaction in the workplace after engaging in OCB more frequently. The initial value of expected satisfaction is set as zero. The following integral function is to represent the expected satisfaction:


$$\begin{array}{l} Expected\;Satisfaction\\ =\text{INTEG}(OCB^{*}Exchange\;Orientation,0). \end{array}$$

## Simulating the Dynamic Computational Model

### The Evaluation of Dynamic Computational Model

**FIGURE 4:**
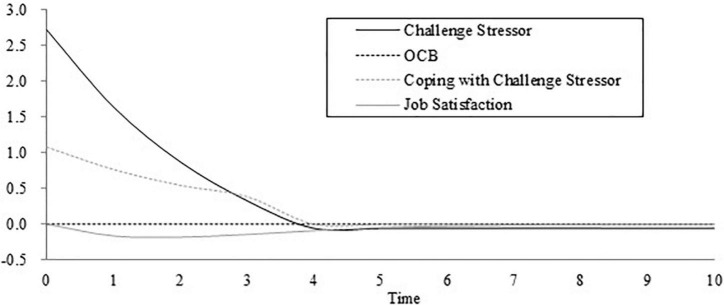
The initial stimulation result of the dynamic computational model.

Unless specified, we set the initial time as 0, the final time as 100, the time step as 1, the unit of time as the day, and the integration type as Euler. We assessed the validity of the dynamic computational model before the next step. An initial assessment is whether the model can produce the phenomenon it is purported to explain (Davis et al., 2007; Vancouver et al., 2014). We simulated the model with the initial parameter and the results are shown in Figure 4. Figure 4 describes the change in challenge stressors, job satisfaction, OCBs, and coping with challenge stressors over time.

**FIGURE 5:**
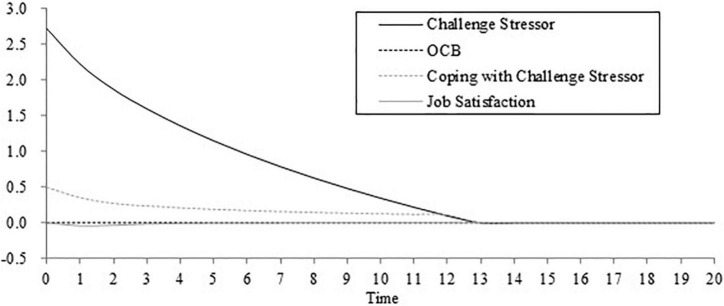
The revised stimulation result of the dynamic computational model (expected challenge stress = 2, expected satisfaction = 2).

As shown in Figure 4, coping with challenge stressors gradually decreases over time and becomes zero on the fourth day. This result is consistent with our assumption that challenge stressors will decrease because of employees’ coping behavior. When the discrepancy between perceived challenge stress and expected challenge stress is narrowed down, the subsequent coping behavior will be reduced. Figure 4 also shows that job satisfaction tends to be a U shape over time. In the default model, the importance of challenge appraisal (0.23) is lower than the importance of strain (0.29). As a result, job satisfaction tends to decline in the initial stage. However, job satisfaction would not keep going down because it will return to the initial equilibrium level after a period of time. As shown in Figure 4, job satisfaction is always lower than the expected satisfaction. Hence, employees do not produce felt obligation and OCB stays at zero. In addition, it is less likely that employees do not expect stress (i.e., the expected challenge stress = 0) and job satisfaction (i.e., the expected job satisfaction = 0). Then, we set these two parameters as two values that are above zero. However, we found that the model is not sensitive to these two parameters after amounts of simulations. Both the expected challenge stress and expected satisfaction are set as 2 and Figure 5 shows the curves within 20 days. Challenge stressors, OCBs, coping with challenge stressors, and job satisfaction change over time in a similar way to that in Figure 5. Due to the increase of expected challenge stress, employees’ effort of coping with challenge stressors is also low under the condition that perceived challenge stress is lower than the expected challenge stress. In the meantime, the change in job satisfaction is not obvious because of the narrowed discrepancy between perceived challenge stress and the expected challenge stress. All these results can explain the corresponding theory and phenomenon. Therefore, we believe that our dynamic computational model is valid.

### Simulation Experiment

**FIGURE 6:**
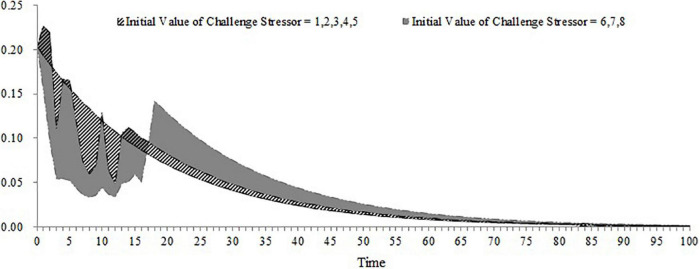
The dynamic change of organizational citizenship behavior (initial equilibrium level > expected satisfaction; importance of challenge appraisal > importance of strain).

**FIGURE 7:**
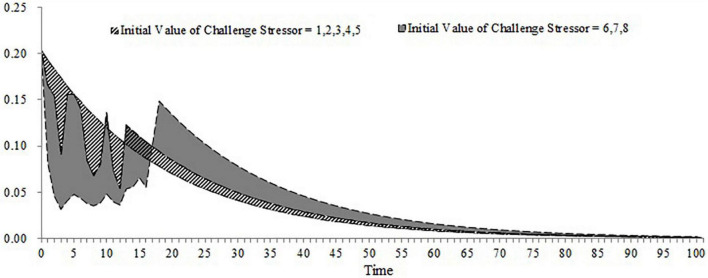
The dynamic change of organizational citizenship behavior (initial equilibrium level > expected satisfaction; importance of challenge appraisal < importance of strain).

**FIGURE 8:**
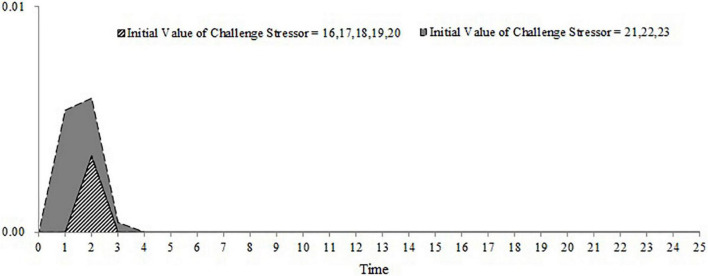
The dynamic change of organizational citizenship behavior (initial equilibrium level < expected satisfaction; importance of challenge appraisal > importance of strain).

This study mainly focuses on the dynamic change of OCB over time under the challenge stressor conditions. According to the expression of OCB, OCB is determined by the felt obligation, which only occurs when job satisfaction is higher than the expected satisfaction. There are two ways to observe the dynamic OCB over time. First, the initial equilibrium level of job satisfaction should be larger than expected satisfaction. Second, the importance of challenge appraisal should be higher than the importance of strain. Therefore, this study clarifies four situations and selects appropriate values in accordance with the situation (see Table 1). Then, we observed the dynamic change of OCB by revising the initial value of the challenge stressor. Figure 6 shows the dynamic change of OCB under the first condition when the initial equilibrium level of job satisfaction is higher than the expected satisfaction and the importance of challenge appraisal is higher than the importance of strain. When the challenge stressor is equal to or lower than the expected challenge stress, OCB tends to decline along with time, and the dynamic change of OCB will occur because employees’ job satisfaction exceeds their expected satisfaction. When the challenge stressor is lower than or equal to 5, OCB tends to be an M shape over time. In the early stage, employees’ OCB increases within a short time because employees have enough resources to engage in OCB within 1 day while coping with challenge stressors. Subsequently, employees will prioritize coping with challenge stressors and conduct less OCB because of the limited resource. Only when challenge stressor has been weakened does OCB increase again. Finally, OCB decreases because of the narrowed discrepancy between job satisfaction and expected satisfaction. When the challenge stressor is above 6, OCB tends to be an inverted N shape over time. In the early stage, OCB tends to decrease because of the limited resource. When employees remove the threat of challenge stressors, OCB increases because of the restored resource. In the late stage, OCB decreases due to the narrowed discrepancy between job satisfaction and expected satisfaction. Figure 7 shows the dynamic change of OCB under the second condition when the initial equilibrium level of job satisfaction is higher than the expected satisfaction and the importance of challenge appraisal is lower than the importance of strain. When the challenge stressor is equal to or lower than the expected challenge stress, OCB tends to decline over time and the explanation of this curve is the same as that in the first situation. When the challenge stressor is larger than the expected challenge stress, OCB tends to be an inverted N-shape over time. The explanation of this curve is the same as that when the challenge stressor is above 6 in the first situation. Figure 8 shows the dynamic change of OCB under the third condition when the initial equilibrium level of job satisfaction is lower than the expected satisfaction and the importance of challenge appraisal is higher than the importance of strain. As shown in Figure 8, only when the challenge stressor is large enough (i.e., *challenge stressor* = 18), employees’ OCB tends to be an inverted U shape in the early stage and the change of OCB is rapid.Finally, OCB is unlikely to occur under the fourth condition when the initial equilibrium level of job satisfaction is lower than the expected satisfaction and the importance of challenge appraisal is lower than the importance of strain. Hence, this study would not analyze the fourth condition in the following simulation.

**TABLE 1:** Conditions and parameter settings.

No.	Condition	Initial equilibrium level	Importance of challenge appraisal
1	Initial Equilibrium Level > Expected Satisfaction = 2 Importance of Challenge Appraisal > Importance of Strain = 0.29	4	0.4
2	Initial Equilibrium Level > Expected Satisfaction = 2 Importance of Challenge Appraisal < Importance of Strain = 0.29	4	0.2
3	Initial Equilibrium Level < Expected Satisfaction = 2 Importance of Challenge Appraisal > Importance of Strain = 0.29	0	0.4
4	Initial Equilibrium Level < Expected Satisfaction = 2 Importance of Challenge Appraisal < Importance of Strain = 0.29	0	0.2

The above analysis demonstrates the dynamic change of OCB without adding any new work tasks. However, employees’ work tasks are unlikely to be static. They might take on new assignments at set intervals. To identify how the dynamic characteristics of the new task influence the accumulation of OCB within a period, we assumed that the new task would be assigned at a fixed frequency and workload. We added the variable of “Task Increment” and changed the expression of challenge stressor into “Challenge Stressor = INTEG(Task Increment-*Coping with Challenge Stressor*, 0).” This study adopted the multiple pulse function “PULSE TRAIN^4^ (*Start*, *Duration*, *Repeattime*, *End*)” to represent task increment. Specifically, this study observed the accumulation of OCB from the 1st to the 100th day. The workload of the new task is the amount of work an employee is assigned each time, and the frequency of the new task refers to the number of times assigned a new task within 100 days. Therefore, we used the following expression to represent task increment:


$$\begin{array}{l} Task\;Increment=Work\;load\;of\;New\;Task^{*}\text{PULSE TRAIN}\\ \left(1,\;1,\;100/Frequency\;of\;the\;new\;task,\;100\right) \end{array}$$

**FIGURE 9:**
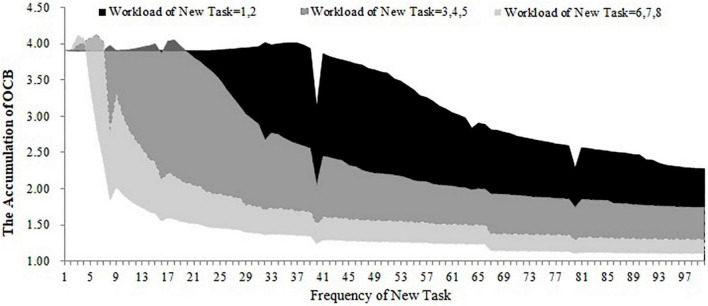
The accumulation of OCB within 100 days when the workload and frequency of new task is at different levels (initial equilibrium level > expected satisfaction; importance of challenge appraisal > importance of strain).

**FIGURE 10:**
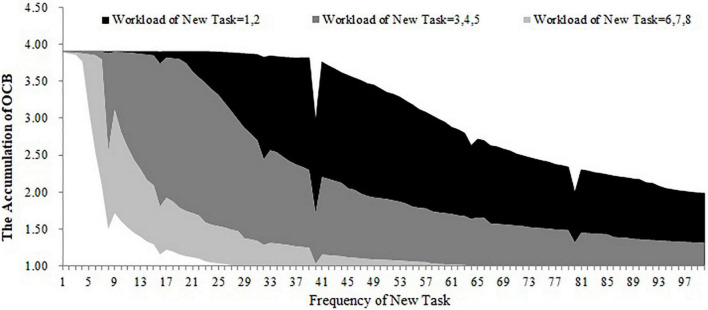
The accumulation of OCB within 100 days when the workload and frequency of new task is at different levels (initial equilibrium level > expected satisfaction; importance of challenge appraisal < importance of strain).

**FIGURE 11:**
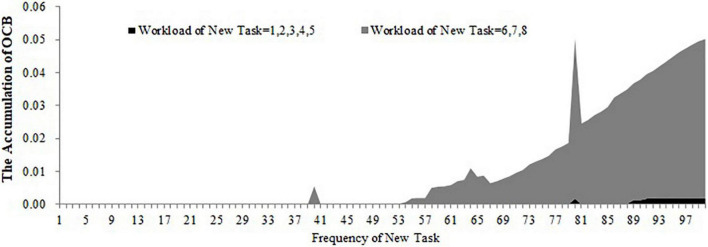
The accumulation of OCB within 100 days when the workload and frequency of new task is at different levels (initial equilibrium level < expected satisfaction; importance of challenge appraisal > importance of strain).

For the first, second, and third conditions, we stimulated the accumulation of OCB within 100 days when the workload and frequency of the new task are at different levels (the workload of the new task ranges from 1 to 8 and the frequency of the new task ranges from 1 to 100). The results are shown in Figures 9–11. As shown in Figure 9, the accumulation of OCB will rise to a peak point and then decline with the increase of the frequency of the new task when the initial equilibrium level of job satisfaction is higher than the expected satisfaction and the importance of challenge appraisal is higher than the importance of strain. Furthermore, when the workload of the new task is higher, the optimal frequency that makes the accumulation of OCB to reach the peak point is smaller and the accumulation of OCB declines faster with the increase of the frequency of the new task. As shown in Figure 10, the accumulation of OCB tends to decline with the increase of the frequency of the new task when the initial equilibrium level of job satisfaction is above the expected satisfaction and the importance of challenge appraisal is below the importance of strain. When the workload of the new task is higher, the accumulation of OCB decreases faster with the increase of the frequency of the new task. Figure 11 describes the accumulation of OCB when the initial equilibrium level of job satisfaction is below the expected satisfaction and the importance of challenge appraisal is above the importance of strain. As shown in Figure 11, the accumulation of OCB stays at zero when the workload of the new task is less than 5. When the workload of the new task is larger than 5, the accumulation of OCB stays at zero when the frequency of the new task is low and then rises with the increase of the frequency of the new task. These results indicate that OCB is not only influenced by the initial value of the challenge stressor but also by the workload and frequency of the new task.

## Discussion

### Theoretical Implications

The main contribution of this study lies in constructing a dynamic computational model of challenge stressors influencing OCB. Although previous research have offered evidence for the dynamic nature of OCB (Smith et al., 2020; Lowery et al., 2021; Parke et al., 2021), we have not yet understood how OCB changes over time under the condition of challenge stressors. Our study fills this gap. Furthermore, this study identifies that the importance of challenge appraisal has a temporary effect on improving OCB and that the initial equilibrium level of job satisfaction has an enduring effect on increasing OCB. By regulating the workload and frequency of assigned new tasks, this study finds the optimal approach to allocate tasks that can increase the accumulation of OCB. This study will discuss the theoretical and practical implications in the following section. This study extends the dynamic computational theory to organizational stress literature and enriches the cybernetic theory of stress. Most stress theories can be explained from the perspective of cybernetics (e.g., Cummings and Gooper, 1979; Edwards, 1992). However, only Vancouver and Weinhardt (2012) applied the dynamic computational model to stress theory and explored the dynamic relationship among the external environment, coping behavior, and job satisfaction. Hence, this study is another attempt of applying the dynamic computational model to stress theory. In addition, Vancouver and Weinhardt’s (2012) model is too simple and overlooks the cognitive appraisal process of stress as well as the effect of OCB during the feedback loop of coping with stress. By accounting for both of these two points, we developed a more nuanced dynamic computational model regarding the process of coping with challenge stressors. By changing the initial challenge stressor and stimulating the dynamic computational model, this study uncovers the role of both the importance of challenge appraisal and the initial equilibrium level of job satisfaction in promoting OCB. The results of the simulation show that challenge stressor is more likely to encourage OCB when employees focus more on the challenge appraisal than the psychological strain or when the initial equilibrium level of job satisfaction is above the expected satisfaction. In contrast, when employees’ challenge appraisal is lower than feeling strain and when the initial equilibrium level of job satisfaction is lower than the expected satisfaction, OCB is less likely to occur regardless of the challenge stressor. To some extent, these results might explain the inconsistent findings in previous studies. This study highlights the role of challenge appraisal and job satisfaction from a dynamic perspective. According to Figure 8, OCB tends to increase rapidly and decrease fast in the early time when the importance of challenge appraisal and challenge stressor is high enough. Hence, we propose that challenge appraisal has a temporarily boosting effect on OCB under the condition of challenge stressor. Comparing Figures 6–8, we acknowledge that employees may be engaging in OCB for a long time when the initial equilibrium level of job satisfaction is higher than the expected satisfaction. It indicates that the positive role of job satisfaction on OCB is enduring under the condition of challenge stressors. This study contributes to identifying the impact of the workload and frequency of assigned new tasks on the accumulation of OCB within a period. The results demonstrate that the optimal frequency of a new task (i.e., the frequency that can maximize the accumulation of OCB) is negatively related to the workload of the new task when the importance of challenge appraisal is larger than the importance of strain and the initial equilibrium level of job satisfaction is higher than the expected satisfaction. When the importance of challenge appraisal is less than the importance of strain and the initial equilibrium level of job satisfaction is higher than the expected satisfaction, the frequency and workload of the new task have an interactive effect on the accumulation of OCB. Specifically, when the workload of the new task is greater, the accumulation of OCB tends to decline more rapidly with the increase in the frequency of the new task. When the importance of challenge appraisal is higher than the importance of strain and the initial equilibrium level of job satisfaction is lower than the expected satisfaction, the frequency and high workload of the new task have an interactive effect on the accumulation of OCB. When the workload of the new task is higher, the accumulation of OCB will increase more steeply with the increase of the frequency of the new task. As the workload is a kind of challenge stressor (Cavanaugh et al., 2000), these results offer a new perspective to explore the impact of challenge stressor. In the future, scholars should not only focus on the static state of challenge stressors but also on the dynamic change of challenge stressors.

### Limitations and Future Research

This study explores the dynamic features of OCB using the dynamic computational model. However, some limitations should be noted. First, although OCB can be encouraged by different motivations (e.g., Rioux and Penner, 2001), we only considered reciprocated motivation. Future research can establish another dynamic computational model by considering other motivational perspectives. In addition, this study controls limited variables which can influence OCB, and this will lead to low external validity. Hence, future studies could collect data in the workplace and examine our propositions empirically. For example, future research can collect daily challenge stressors and daily OCB across several consecutive days and use these daily data to reexamine our findings.Second, there are many variables and parameters in the dynamic computational model and almost all of the parameters are based on previous literature which might bias the findings. In the future, scholars can measure the variables in the model and estimate the parameters. Despite that many propositions can be obtained by changing the parameters, this study mainly focuses on the importance of challenge appraisal and the initial equilibrium level of job satisfaction. Future studies can examine the potential role of other static variables, like exchange orientation, the importance of coping behavior, and the importance of OCB. Third, this study adopts multiple pulse function to analyze the effect of the frequency and workload of assigned new tasks. However, the multiple pulse function assumes that the tasks are assigned to employees at a fixed number and frequency, which is different from that in the actual workplace. Future research can stimulate our model and observe the accumulation of OCB within 1 year by using the real workload and frequency of assigned new tasks.

### Practical Implications

The practical implications of this study are focused on how to motivate employees’ engagement in OCB while coping with challenge stressors. According to the results of this study, it is not enough to advocate imposing high levels of challenge stressors on employees like previous research (e.g., Cavanaugh et al., 2000; LePine et al., 2005). More importantly, managers should make efforts in the following two approaches. First, when assigning challenging job requirements to employees, managers need to tell employees about the positive linkage between challenge stressors and personal growth. In this way, employees are more likely to produce challenge appraisal and job satisfaction in the face of challenge stressors, thereby increasing OCB. Second, managers should employ effective strategies to improve employees’ overall job satisfaction because OCB is less likely to occur when employees’ overall job satisfaction is lower than expected. In addition, the findings of this study offer suggestions for managers on how to assign tasks to employees. For employees who focus on the beneficial outcomes of challenge stressors and experience high levels of job satisfaction, managers should understand that there is a trade-off between the frequency and workload of assigned new tasks. To maximize OCBs, managers should assign these employees more tasks at a lower frequency or assign these employees fewer tasks at a higher frequency. For employees who give weight to the strain associated with the challenge stressor and experience high levels of job satisfaction, the frequency of assigned new tasks should be kept at lower levels regardless of the workload of the new task. For employees who attach importance to the beneficial outcomes of challenge stressors and experience low levels of job satisfaction, the frequency and workload of the new task should be increased synchronously. Finally, our dynamic computational theory regarding the stressor-OCB relationship helps **employees** understand when their **co-**workers are more likely to engage in OCB. Considering that helping others is a kind of OCB (**Smith et al., 1983**), the findings of this study enable **employees** to know when enlisting the help of colleagues is more likely to get a response if they need help. Specifically, **employees** can seek colleagues for help when challenging job requirements are allocated to these colleagues at the beginning or when these requirements are completed by these colleagues because our theory demonstrated that OCB is higher under these two conditions. This knowledge helps them avoid the embarrassment and helplessness that comes with being denied help.

## Data Availability Statement

The raw data supporting the conclusions of this article will be made available by the authors, without undue reservation.

## Author Contributions

LC and LZ drafted and designed the work. LC and QB constructed the computational model. LC stimulated the model and drafted the manuscript. LZ is the recipient of the fund. LZ and QB critically revised the manuscript. All authors contributed to the article and approved the submitted version.

## Conflict of Interest

The authors declare that the research was conducted in the absence of any commercial or financial relationships that could be construed as a potential conflict of interest.

## Publisher’s Note

All claims expressed in this article are solely those of the authors and do not necessarily represent those of their affiliated organizations, or those of the publisher, the editors and the reviewers. Any product that may be evaluated in this article, or claim that may be made by its manufacturer, is not guaranteed or endorsed by the publisher.

## Footnotes

3 We adopted the daily correlation coefficient between time pressure and challenge appraisald as well as the daily correlation coefficient between learning demands and challenge appraisals in Prem et al.’s (2017) study. The value of 0.23 is the average of these two daily correlation coefficients.

4 In the software of Vensim, the “PULSE TRAIN (Start, Duration, Repeattime, End)” function will return the value of “1,” start at time “Start,” last for interval “Duration,” repeat this pattern every “Repeattime” time, and end at time “End.”

## References

[B1] Bergeron D. M. (2007). The potential paradox of organizational citizenship behavior: good citizens at what cost? *Acad. Manage. Rev.* 32 1078–1095. 10.5465/AMR.2007.26585791

[B2] Blau P. M. (1964). *Exchange and Power in Social Life.* New York, NY: John Wiley.

[B3] Cavanaugh M. A. Boswell W. R. Roehling M. V. Boudreau J. W. (2000). An empirical examination of self-reported work stress among US managers. *J. Appl. Psychol.* 85 65–74. 10.1037//0021-9010.85.1.65 10740957

[B4] Chen Z. X. Zhao C. H. Tu H. (2019). Antecedents of the tipple effect of leadership ostracism: the role of competition and organizational politics. *J. Ind. Eng. /Eng. Man.* 33 215–222. 10.13587/j.cnki.jieem.2019.03.025

[B5] Cropanzano R. Mitchell M. S. (2005). Social exchange theory: an interdisciplinary review. *J. Manage.* 31 874–900.

[B6] Cummings T. G. Gooper G. L. (1979). Cybernetic framework for studying occupational stress. *Hum. Relat.* 32 395–418.

[B7] Davis J. P. Eisenhardt K. M. Bingham C. B. (2007). Developing theory through simulation methods. *Acad. Manage. Rev.* 32 480–499. 10.2307/20159312

[B8] Eatough E. M. Chang C. H. Miloslavic S. A. Johnson R. E. (2011). Relationships of role stressors with organizational citizenship behavior: a meta-analysis. *J. Appl. Psychol.* 96 619–632. 10.1037/a0021887 21244128

[B9] Edwards J. R. (1992). A cybernetic theory of stress, coping, and well-being in organizations. *Acad. Manage. Rev.* 17 238–274. 10.2307/258772

[B10] Eisenberger R. Armeli S. Rexwinkel B. Lynch P. D. Rhoades L. (2001). Reciprocation of perceived organizational support. *J. Appl. Psychol.* 86 42–51. 10.1037/0021-9010.86.1.42 11302232

[B11] Gong Y. Chang S. Cheung S. Y. (2010). High performance work system and collective OCB: a collective social exchange perspective. *Hum. Resour. Manag. J.* 20 119–137. 10.1111/j.1748-8583.2010.00123.x

[B12] Haldorai K. Kim W. G. Phetvaroon K. (2022). Beyond the bend: the curvilinear effect of challenge stressors on work attitudes and behaviors. *Tour. Manage.* 90:104482. 10.1016/j.tourman.2021.104482

[B13] Hobfoll S. E. (1989). Conservation of resources: a new attempt at conceptualizing stress. *Am. Psychol.* 44 513–524. 10.1037/0003-066X.44.3.513 2648906

[B14] Ilies R. Fulmer I. S. Spitzmuller M. Johnson M. D. (2009). Personality and citizenship behavior: the mediating role of job satisfaction. *J. Appl. Psychol.* 94 945–959. 10.1037/a0013329 19594236

[B15] Jex S. M. (1998). *Stress and Job Performance: Theory, Research, and Implications for Managerial Practice.* Thousand Oaks, CA: Sage.

[B16] Khliefat A. Chen H. Ayoun B. Eyoun K. (2021). The impact of the challenge and hindrance stress on hotel employees interpersonal citizenship behaviors: psychological capital as a moderator. *Int. J. Hosp. Manag.* 94:102886. 10.1016/j.ijhm.2021.102886

[B17] Lam C. F. Liang J. Ashford S. J. Lee C. (2015). Job insecurity and organizational citizenship behavior: exploring curvilinear and moderated relationships. *J. Appl. Psychol*. 100 499–510. 10.1037/a0038659 25602119

[B18] Landy F. J. (1978). An opponent process theory of job satisfaction. *J. Appl. Psychol.* 63 533–547. 10.1037/0021-9010.63.5.533

[B19] LePine J. A. LePine M. A. Jackson C. L. (2004). Challenge and hindrance stress: relationships with exhaustion, motivation to learn, and learning performance. *J. Appl. Psychol.* 89 883–891. 10.1037/0021-9010.89.5.883 15506867

[B20] LePine J. A. Podsakoff N. P. LePine M. A. (2005). A meta-analytic test of the challenge stressor-hindrance stressor framework: an explanation for inconsistent relationships among stressors and performance. *Acad. Manage. J.* 48 764–775. 10.5465/AMJ.2005.18803921

[B21] Lord R. G. Hanges P. J. (1987). A control system model of organizational motivation: theoretical development and applied implications. *Behav. Sci.* 32 161–178. 10.1002/bs.3830320302

[B22] Lowery M. R. Clark M. A. Carter N. T. (2021). The balancing act of performance: psychometric networks and the causal interplay of organizational citizenship and counterproductive work behaviors. *J. Vocat. Behav.* 125:103527. 10.1016/j.jvb.2020.103527

[B23] Ma E. Qu H. (2011). Social exchanges as motivators of hotel employees’ organizational citizenship behavior: the proposition and application of a new three-dimensional framework. *Int. J. Hosp. Manag.* 30 680–688. 10.1016/j.ijhm.2010.12.003

[B24] Ma J. Peng Y. Wu B. (2021). Challenging or hindering? The roles of goal orientation and cognitive appraisal in stressor-performance relationships. *J. Organ. Behav.* 42 388–406. 10.1002/job.2503

[B25] Mazzola J. J. Ryan D. (2019). Should we be “challenging” employees? A critical review and meta-analysis of the challenge-hindrance model of stress. *J. Organ. Behav.* 40 949–961. 10.1002/job.2412

[B26] McCauley C. D. Ruderman M. N. Ohlott P. J. Morrow J. E. (1994). Assessing the developmental components of managerial jobs. *J. Appl. Psychol.* 79 544–560. 10.1037/0021-9010.79.4.544

[B27] Mischel W. (1973). Toward a cognitive social learning reconceptualization of personality. *Psychol. Rev.* 80 252–283. 10.1037/h0035002 4721473

[B28] Montani F. Dagenais-Desmarais V. (2018). Unravelling the relationship between role overload and organizational citizenship behaviour: a test of mediating and moderating effects. *Eur. Manag. J.* 36 757–768. 10.1016/j.emj.2018.03.001

[B29] Murstein B. I. Cerreto M. Mac Donald M. G. (1977). A theory and investigation of the effect of exchange-orientation on marriage and friendship. *J. Marriage Fam.* 39 543–548.

[B30] Parke M. R. Tangirala S. Hussain I. (2021). Creating organizational citizens: how and when supervisor-versus peer-led role interventions change organizational citizenship behavior. *J. Appl. Psychol.* 106 1714–1733. 10.1037/apl0000848 33090860

[B31] Pletzer J. L. (2021). Why older employees engage in less counterproductive work behavior and in more organizational citizenship behavior: examining the role of the HEXACO personality traits. *Pers. Indiv. Differ.* 173:110550. 10.1016/j.paid.2020.110550

[B32] Podsakoff N. P. LePine J. A. LePine M. A. (2007). Differential challenge stressor-hindrance stressor relationships with job attitudes, turnover intentions, turnover, and withdrawal behavior: a meta-analysis. *J. Appl. Psychol.* 92 438–454. 10.1037/0021-9010.92.2.438 17371090

[B33] Podsakoff P. M. MacKenzie S. B. (1997). Impact of organizational citizenship behavior on organizational performance: a review and suggestion for future research. *Hum. Perform.* 10 133–151. 10.1207/s15327043hup1002_5 33486653

[B34] Pooja A. A. De Clercq D. Belausteguigoitia I. (2016). Job stressors and organizational citizenship behavior: the roles of organizational commitment and social interaction. *Hum. Resour. Dev. Q.* 27 373–405. 10.1002/hrdq.21258

[B35] Prem R. Ohly S. Kubicek B. Korunka C. (2017). Thriving on challenge stressors? Exploring time pressure and learning demands as antecedents of thriving at work. *J. Organ. Behav.* 38 108–123. 10.1002/job.2115 28133415 PMC5244684

[B36] Rioux S. M. Penner L. A. (2001). The causes of organizational citizenship behavior: a motivational analysis. *J. Appl. Psychol.* 86 1306–1314. 10.1037/0021-9010.86.6.1306 11768072

[B37] Roch S. G. Shannon C. E. Martin J. J. Swiderski D. Agosta J. P. Shanock L. R. (2019). Role of employee felt obligation and endorsement of the just world hypothesis: a social exchange theory investigation in an organizational justice context. *J. Appl. Soc. Psychol.* 49 213–225. 10.1111/jasp.12578

[B38] Rodell J. B. Judge T. A. (2009). Can “good” stressors spark “bad” behaviors? The mediating role of emotions in links of challenge and hindrance stressors with citizenship and counterproductive behaviors. *J. Appl. Psychol.* 94 1438–1451. 10.1037/a0016752 19916654

[B39] Rogosa D. (1988). “Myths about longitudinal research,” in *Methodological Issues in Aging Research*, eds Schaie K. W. Campbell R. T. Meredith W. Rawlings S. C. (New York, NY: Springer), 171–209.

[B40] Rosen C. C. Dimotakis N. Cole M. S. Taylor S. G. Simon L. S. Smith T. A. (2020). When challenges hinder: an investigation of when and how challenge stressors impact employee outcomes. *J. Appl. Psychol.* 105 1181–1206. 10.1037/apl0000483 31999135

[B41] Skinner N. Brewer N. (2002). The dynamics of threat and challenge appraisals prior to successful achievement events. *J. Pers. Soc. Psychol.* 83 678–692. 10.1037/0022-3514.83.3.678 12219862

[B42] Smith C. A. Organ D. W. Near J. P. (1983). Organizational citizenship behavior: its nature and antecedents. *J. Appl. Psychol.* 68 653–663. 10.1037/0021-9010.68.4.653

[B43] Smith R. W. Kim Y. J. Carter N. T. (2020). Does it matter where you’re helpful? Organizational citizenship behavior from work and home. *J. Occup. Health Psychol*. 25 450–468. 10.1037/ocp0000181 32271040 PMC8363146

[B44] Spector P. E. (1997). *Job Satisfaction: Application, Assessment, Causes and Consequences.* Thousand Oaks, CA: Sage Publications, Inc.

[B45] Thoresen C. J. Kaplan S. A. Barsky A. P. Warren C. R. de Chermont K. (2003). The affective underpinnings of job perceptions and attitudes: a meta-analytic review and integration. *Psychol. Bull.* 129 914–945. 10.1037/0033-2909.129.6.914 14599288

[B46] Van Dyne L. Graham J. W. Dienesch R. M. (1994). Organizational citizenship behavior: construct redefinition, measurement, and validation. *Acad. Manage. J.* 37 765–802. 10.2307/256600

[B47] Vancouver J. B. Weinhardt J. M. (2012). Modeling the mind and the milieu computational modeling for micro-level organizational researchers. *Organ. Res. Methods* 15 602–623. 10.1177/1094428112449655

[B48] Vancouver J. B. Tamanini K. B. Yoder R. J. (2010). Using dynamic computational models to reconnect theory and research: socialization by the proactive newcomer as example. *J. Manage.* 36 764–793. 10.1177/0149206308321550

[B49] Vancouver J. B. Weinhardt J. M. Vigo R. (2014). Change one can believe in: Adding learning to computational models of self-regulation. *Organ. Behav. Hum. Dec*. 124, 56–74. 10.1016/j.obhdp.2013.12.002

[B50] Wallace J. C. Edwards B. D. Arnold T. Frazier M. L. Finch D. M. (2009). Work stressors, role-based performance, and the moderating influence of organizational support. *J. Appl. Psychol.* 94 254–262. 10.1037/a0013090 19186910

[B51] Webster J. R. Beehr T. A. Love K. (2011). Extending the challenge-hindrance model of occupational stress: the role of appraisal. *J. Vocat. Behav.* 79 505–516. 10.1016/j.jvb.2011.02.001

[B52] Wells D. L. Moorman R. H. Werner J. M. (2007). The impact of the perceived purpose of electronic performance monitoring on an array of attitudinal variables. *Hum. Resour. Dev. Q.* 18 121–138. 10.1002/hrdq.1194

[B53] Zhang Y. L. Lu C. Q. (2009). Challenge stressor-hindrance stressor and employees’ work-related attitudes, and behaviors: the moderating effects of general self-efficacy. *Acta Psychol. Sin.* 41 35–43. 10.3724/sp.j.1041.2009.00501

[B54] Zhang Y. LePine J. A. Buckman B. R. Wei F. (2014). It’s not fair…or is it? The role of justice and leadership in explaining work stressor-job performance relationships. *Acad. Manage. J.* 57 675–697. 10.5465/amj.2011.1110

